# A Correlation Study of Plasma and Breast Milk Retinol Concentrations in Breastfeeding Women in China

**DOI:** 10.3390/nu15245085

**Published:** 2023-12-12

**Authors:** Jing Qin, Yubo Zhou, Hongtian Li, Ying Meng, Sherry A. Tanumihardjo, Jianmeng Liu

**Affiliations:** 1 National Health Commission Key Laboratory of Reproductive Health/Institute of Reproductive and Child Health, Peking University Health Science Center, Beijing 100191, China; qinjing_2014@163.com (J.Q.); liht@bjmu.edu.cn (H.L.); mengying@pku.edu.cn (Y.M.); 2 Department of Epidemiology and Biostatistics, School of Public Health, Peking University Health Science Center, Beijing 100191, China; 3 Department of Nutritional Sciences, University of Wisconsin-Madison, Madison, WI 53706, USA; sherry@nutrisci.wisc.edu; 4 Center for Intelligent Public Health, Institute for Artificial Intelligence, Peking University, Beijing 100191, China

**Keywords:** breast milk, plasma, retinol, vitamin A

## Abstract

Retinol in breast milk is related to plasma concentration among breastfeeding women, but the linear or curvilinear relationships between the two remains unclear. We conducted a cross-sectional study in 403 Chinese breastfeeding women at 42 ± 7 days postpartum. Plasma and breast milk samples were assayed using high performance liquid chromatography to determine the concentration of retinol. Partial Spearman correlation and multivariable fractional polynomial regression were used to examine the relationships between the two retinol concentrations and between plasma retinol concentration and milk-to-plasma (M/P) retinol. The median (interquartile range, IQR) of the retinol concentration in the plasma was 1.39 (1.21, 1.63) μmol/L and 1.15 (0.83, 1.49) μmol/L in the breast milk, respectively. The partial correlation coefficient between them was 0.17 (*p* < 0.01). A linear relationship was observed with an adjusted regression coefficient of 0.34 (95% CI: 0.19, 0.49). The relationship between the plasma retinol and M/P ratio was nonlinear and segmented at 1.00 μmol/L of plasma retinol. The regression coefficients, below and above the segmented point, were −1.69 (95% CI: −2.75, −0.62) and −0.29 (95% CI: −0.42, −0.16), respectively. Plasma and breast milk retinol were positively correlated, whereas women with a low concentration of plasma retinol showed a stronger capacity of transferring retinol to breast milk.

## 1. Introduction

Vitamin A (VA) is an essential nutrient for cell growth and differentiation as well as for maintaining vision, immunity, and reproduction [1]. VA deficiency remains a challenge in developing countries, particularly in vulnerable populations such as breastfeeding women and infants [2]. Breastfeeding women need adequate VA to meet an increased requirement for themselves, as well as their infant. Generally, infants are born with inadequate VA stores and have to rely on maternal breast milk to acquire VA in early life, especially given that the World Health Organization (WHO) has recommended exclusive breastfeeding for infants under 6 months [3]. VA concentration in breast milk rests on maternal dietary intake and plasma VA concentrations [4]. Dietary VA can be directly transferred to the mammary gland via chylomicron [5], and maternal plasma retinol is bound to a retinol-binding protein and then transferred to the mammary gland through a receptor-mediated process or by free diffusion [6].

Previous studies have explored the relationship between VA concentrations in plasma and breast milk but reported inconsistent results. There have been six studies [7,8,9,10,11,12] that have reported a positive relation between maternal plasma and mature milk, whereas another three studies [13,14,15] showed no significant relation between them. There have been four studies [16,17,18,19] that showed no relationship between plasma and colostrum VA, which is likely due to the studies’ limited sample sizes (*n* = 33 to 103). In addition, none of these previous studies explored the linear or curvilinear relationships between plasma and breast milk VA concentrations. Furthermore, such data for the Chinese population is still lacking. The relationships are merited to be further evaluated in breastfeeding women with a larger sample size, since a sufficient understanding of the relationships is crucial for optimizing feeding practices of infants to benefit their growth and development.

Therefore, we conducted a large-scale cross-sectional study in Chinese breastfeeding women at 42 ± 7 days postpartum to examine the relationships between maternal plasma and breast milk retinol concentrations, and to examine whether the relationships differed by regions with distinct dietary patterns. We further examined the relationship of plasma retinol concentration with the milk-to-plasma (M/P) ratio that likely reflects a retinol-transferring profile from plasma into breast milk.

## 2. Materials and Methods

### 2.1. Subjects

This cross-sectional study was approved by the Institutional Review Board/Human Subjects Committee at Peking University Health Science Center (IRB00001052-14012; date of approval: 22 April 2014), and informed consent forms were signed by all participants. The study was conducted at four hospitals located in Weihai (central), Yueyang (southern), and Baotou (northern) cities of China between May and July 2014, with the original aim to assess the docosahexaenoic acid status in Chinese pregnant and breastfeeding women, as described elsewhere [20]. The present study used the data and samples from the 416 breastfeeding women recruited at 42 ± 7 days of postpartum. The inclusion criteria of these women were as follows: (1) generally healthy, (2) 18–35 years old, (3) local permanent residents, (4) having had a singleton delivery, and (5) currently breastfeeding. The exclusion criteria were as follows: (1) having been diagnosed with severe cardiovascular, metabolic or renal diseases, or mental disorders, (2) allergic to aquatic food, or (3) participated in other research projects in the past 30 days. Among the 416 initially recruited breastfeeding women, 9 were excluded due to maternal age greater than 35 years (*n* = 6) or over 49 days postpartum (*n* = 3), and 4 due to retinol concentration outliers which were defined in the following statistical analysis section. Finally, 403 women were included in the present analysis (Supplementary Figure S1).

### 2.2. Data and Sample Collection

Information about maternal and infantile characteristics was collected by trained obstetricians or nurses using a structured questionnaire. The detailed information included maternal age, ethnicity (Han and others), educational level (college or above, high school, and middle school or less), height, weight, parity (primiparous and multiparous), gestational age, delivery modes (vaginal delivery and cesarean section), and breastfeeding practice (exclusive breastfeeding and partial breastfeeding), as well as sex (male and female) and birth weight of infants.

About 5 mL of fasting venous blood and 10 mL of full breast milk were collected for each woman in the morning (10 ± 2 AM). The detailed collection and processing procedures have been described elsewhere [20]. In brief, the blood samples were collected into ethylenediaminetetraacetic acid-containing tubes and held in a refrigerator at 5 °C for about 30 min before being separated to obtain plasma aliquots. Full breast milk samples were collected into a sterile container manually or using a breast pump from a non-feeding breast that has not been used to feed an infant for ≥1 h. Both the plasma and breast milk samples were temporarily stored at local hospitals at −20 °C for about 10 days, then they were transported to the National Health Commission Key Laboratory of Reproductive Health at Peking University Health Science Center and stored at −80 °C until analysis.

### 2.3. Sample Analysis

Plasma and breast milk retinol analyses were performed at the National Health Commission Key Laboratory of Reproductive Health using high performance liquid chromatography (HPLC, Waters Alliance 2690 system) equipped with a UV/visible detector (Waters UV detector model 2468) (Waters Chromatography Division, Milford, MA, USA) and a C18 reversed-phase column (3.5 μm, 4.6 × 150 nm; Waters, Milford, MA, USA). All samples were assayed under yellow lights.

The plasma retinol concentration was determined according to the method recommended by the Centers for Disease Control of the U.S. [21]. After thawing and gentle mixing, 25 μL of plasma was mixed with 50 μL of ethanol containing the internal standard retinyl acetate (Sigma Chemical Co., St. Louis, MO, USA), then 125 μL of acetonitrile was added for extraction. The mixture was mixed and centrifuged at 1000 rpm/min for 3 min at room temperature, then the supernatant was transferred to a sealed vial for injection. The chromatogram was developed in isocratic elution with a 78:22 acetonitrile/deionized water (0.1% triethylamine) mobile phase and 0.9 mL/min flow rate. The peak response of retinol was measured at 325 nm. The standard curve was based on five points ranging from 0.02 μmol/L to 3.49 μmol/L. The accuracy of the sample measurement was assessed by analyzing 3 levels of the quality-control samples embedded into each batch of samples. Samples would be retested if two or more quality-control results were outside the mean ± 2 standard deviations (SDs) or if any of the quality-control results were outside the mean ± 3 SDs.

The breast milk retinol concentration was determined according to a method adapted from Tanumihardjo [22]. After thawing and gentle mixing, 0.5 mL of homogenized breast milk was mixed with 0.75 mL of ethanol, 80 μL of internal standard C23-apo-carotenol [23], and 0.4 mL of 50%-KOH solution in a 5 mL screw-top vial. The mixture was placed in a water bath at 45 °C for 1 h and mixed every 15 min. Then 0.75 mL of hexane was added for extraction. After mixing and centrifuging at 3000 rpm/min for 1 min at room temperature, the supernatant was transferred to a glass tube. The extraction was repeated 3 times, and the combined extracts were evaporated under nitrogen. The residue was redissolved in 100 μL of 75:25 methanol: dichloromethane, and 25 μL was injected into the HPLC system. The chromatogram was developed in an isocratic elution with 95:5 methanol: deionized water (0.5‰ triethylamine) mobile phase and 1.0 mL/min flow rate. The peak response of retinol was measured at 340 nm. The peak areas were calculated with correction for the extraction efficiency, which was calculated by dividing the integrator area obtained for the internal standard in the sample by the expected area of the equivalent internal standard. The standard curve was obtained by measuring 5, 10, 20, and 40 μL of retinol of certain absorbance (approximately 0.324).

### 2.4. Statistical Analysis

The medians (interquartile ranges, IQRs) were presented for plasma and breast milk retinol, due to their non-normal distributions (*p*-values < 0.001 by the Kolmogorov–Smirnov D test). The arithmetic means and SDs were also presented, for comparing with estimates in previous studies. Outliers of retinol concentration were identified as values > 3 IQRs from the 75th percentile: subjects with plasma retinol concentrations > 3.17 μmol/L (*n* = 1) and breast milk retinol concentrations > 3.61 μmol/L (*n* = 4) were excluded. The M/P retinol ratio was calculated as dividing breast milk retinol concentration by plasma retinol concentration to represent the efficiency of plasma retinol transfer to breast milk. The statistical description of the M/P ratio is the same as that of retinol concentration. The statistical differences of retinol concentrations in plasma and breast milk and the M/P ratio across regions were examined by Kruskal–Wallis tests, followed by Bonferroni corrected Mann–Whitney tests for multiple comparisons.

Crude and partial Spearman correlation coefficients were calculated to determine relationships between plasma and breast milk retinol concentrations, and the M/P ratio. The correlation analyses were repeated in subgroups stratified by regions, and the differences in correlation coefficients between any two regions were tested using *t*-tests with Fisher r-to-z transformation, followed by Bonferroni corrected *p*-values. Multivariable fractional polynomial (MFP) regression models [24] were used to examine the potential nonlinear relationship between plasma and breast milk retinol concentrations and the relationship between the plasma retinol concentration and M/P ratio. Segmented regression models [25] were used to: (a) identify breakpoints in the nonlinear relationship between plasma retinol and the M/P ratio, as indicated by the fitted MFP regression model; and (b) further assess the linear correlation between plasma retinol and the M/P ratio in different segments. In the partial Spearman correlation analyses and multivariable regression models, adjusted categorical variables included regions, maternal age (<25, ≥25 to 30, and ≥30 years), ethnicity, education level, body mass index (BMI, calculated as weight in kilograms divided by squared height in meters; <18.5, 18.5 to <25, and ≥25 kg/m^2^), parity, gestational age (<37, 37 to <42, and ≥42 weeks), delivery modes, breastfeeding practice, sex and birth weight of infants (<2500, 2500 to <4000, and ≥4000 g).

Because the relationship between plasma and breast milk retinol is likely affected by maternal dietary intake of VA, we performed stratified analyses to examine the relationships by regions that might reflect different dietary patterns. We added an interaction term between the regions and plasma retinol concentration into the linear regression model to examine whether effects of plasma retinol on the milk varied by geographic regions. Additionally, we conducted subgroup analyses stratified by breastfeeding practices or delivery modes, to assess the robustness of the relationship between plasma retinol concentration and the M/P ratio.

Statistical analyses were performed by using SPSS version 18.0 (Chicago, IL, USA) and R version 3.6.0 (R Development Core Team, Vienna, Austria) software. The statistical significance was set at *p* < 0.05 (two-sided).

## 3. Results

### 3.1. Maternal and Infant Characteristics

The median postpartum days of the breastfeeding women were 42 (IQR, 40, 44) days. Overall, 62.3% of the women were 25–30 years, 85.1% were primiparous, 95.5% had Han ethnicity, 67.0% had college or above education, 91.1% delivered at 37–42 gestational weeks, and 88.8% delivered neonates with birth weights of 2500 to <4000 g. The proportions of the women residing in the central, southern, and northern regions were 33.5%, 33.3%, and 33.3%, respectively. There were significant regional differences in most characteristics except maternal education, parity, gestational age, breastfeeding practice, and birth weight (Table 1).

### 3.2. Relationship between Plasma and Breast Milk Retinol Concentrations

The median (IQR) retinol concentration in plasma was 1.39 (1.21, 1.63) μmol/L, and 1.15 (0.83, 1.49) μmol/L in breast milk. The overall crude correlation coefficient between the two was 0.17 (*p* = 0.005, Table 2).

**Figure 1:**
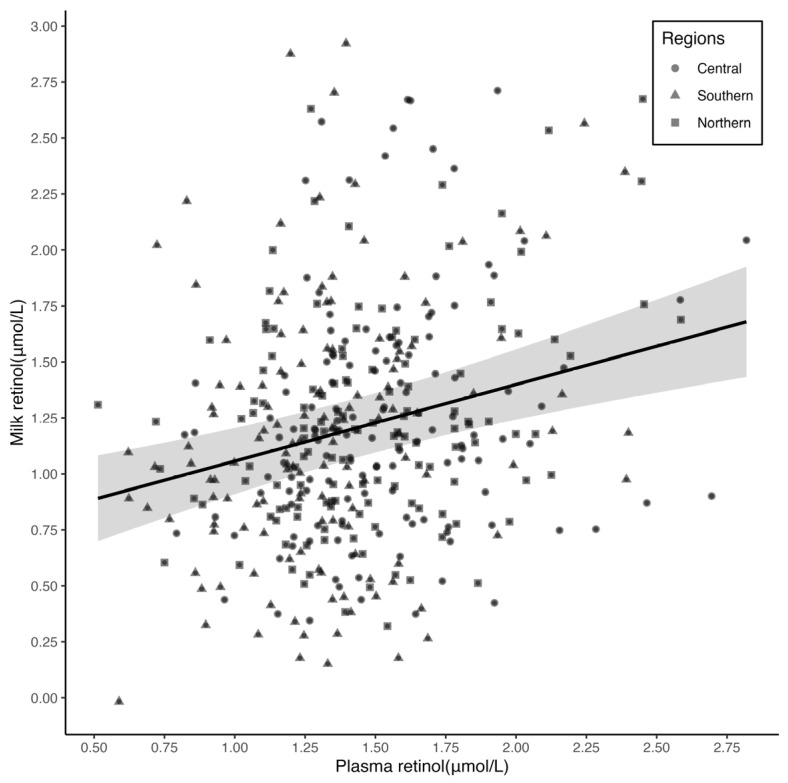
Correlation between plasma and breast milk retinol. Multivariable fractional polynomial (MFP) regression was adjusted for regions, maternal age, ethnicity, education level, BMI, parity, gestational age, delivery modes, breastfeeding practice, sex and birth weight of infants. Solid line represents the effect estimates and the shade represents the 95% confidence intervals.

After multivariable adjustment, the partial Spearman correlation coefficient was 0.17 (*p* = 0.006). In the subgroup analysis stratified by regions, the Spearman correlation coefficients were 0.23 (*p* = 0.012) in central, 0.21 (*p* = 0.017) in southern, and 0.12 (*p* = 0.178) in northern, and did not significantly differ across the regions (*p*-value > 0.05). A linear relationship between plasma and breast milk retinol was observed (the power exponent was 1, and *p* for the test of nonlinearity was 0.879) (Figure 1), with a regression coefficient of 0.34 (95% CI: 0.19,0.49) (Table 3).

### 3.3. Relationship between Plasma Retinol Concentration and M/P Ratio

**Figure 2:**
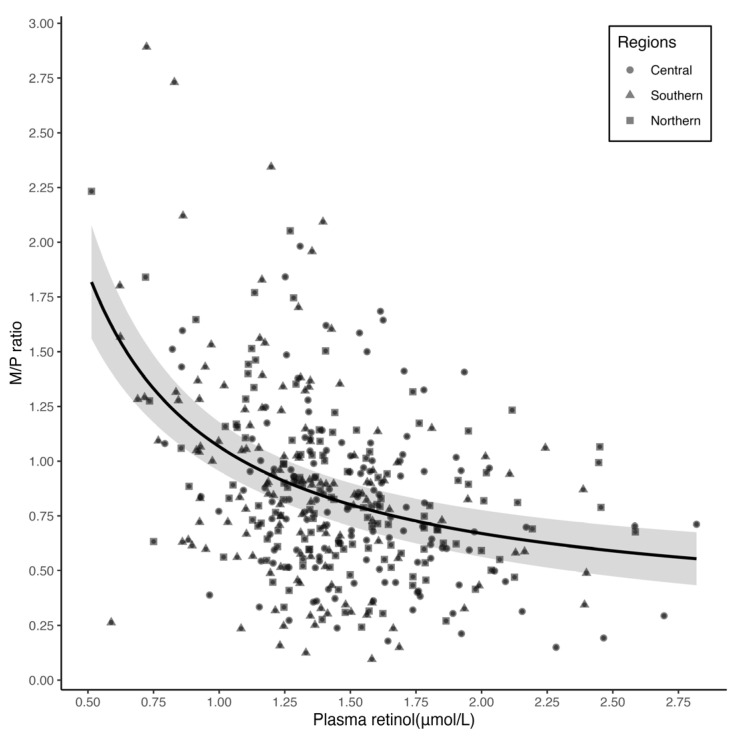
Correlation between plasma retinol and the milk-to-plasma (M/P) ratio. Nonlinear dose–response relationship between plasma retinol and the M/P ratio from MFP, and adjusted for regions, maternal age, ethnicity, education level, BMI, parity, gestational age, delivery modes, breastfeeding practice, sex and birth weight of infants. The M/P ratio decreased by 1.69 (95% CI: 0.62, 2.75) and 0.29 (95% CI: 0.16, 0.42) per 1.00 μmol/L plasma retinol below and above 1.00 μmol/L plasma retinol, respectively, in a segmented linear multivariate model. Solid line represents the effect estimates and the shade represents the 95% confidence intervals.

**Figure 3:**
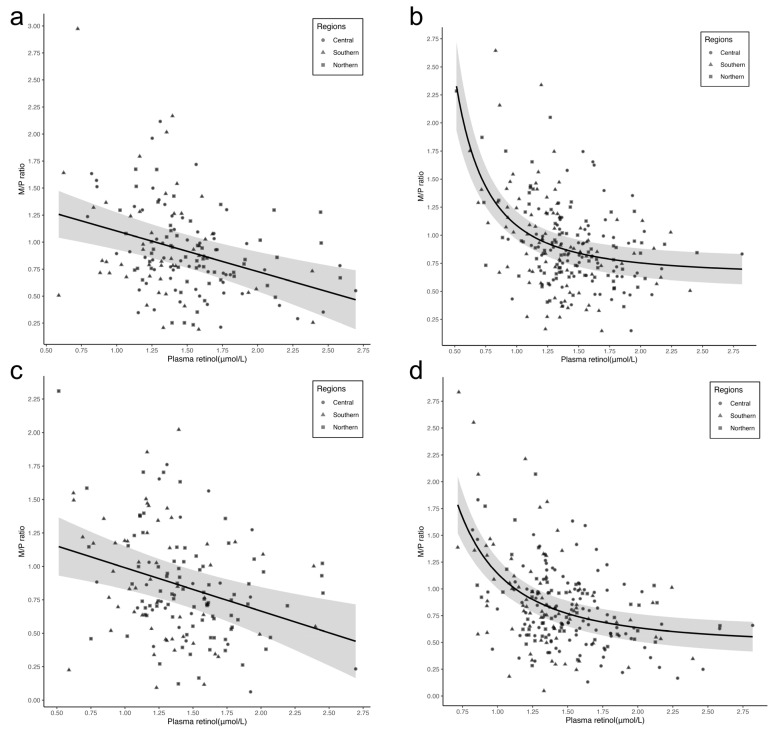
Correlation between plasma retinol and the M/P ratio, stratified by breastfeeding practice and delivery modes. Linear or Nonlinear dose–response relationship between plasma retinol and M/P ratio from MFP, and adjusted for regions, maternal age, ethnicity, education level, BMI, parity, gestational age, delivery modes, breastfeeding practice, sex and birth weight of infants. (**a**) A linear relationship between plasma retinol and the M/P ratio from MFP among partially breastfed women; (**b**) a nonlinear relationship between plasma retinol and the M/P ratio from MFP among exclusively breastfed women; (**c**) a linear relationship between plasma retinol and the M/P ratio from MFP among women who had a cesarean section; and (**d**) a nonlinear relationship between plasma retinol and the M/P ratio from MFP among women who had a vaginal delivery. Solid lines represent the effect estimates and the shade represents the 95% confidence intervals.

The median (IQR) M/P retinol ratio was 0.80 (0.57, 1.12). The overall crude and partial Spearman correlation coefficients between plasma retinol and M/P ratio were −0.34 (*p* < 0.001) and −0.33 (*p* < 0.001), respectively. In subgroup analyses stratified by regions, the Spearman correlation coefficients were −0.25 (*p* = 0.005) in central, −0.33 (*p* < 0.001) in southern, and −0.37 (*p* < 0.001) in northern and did not significantly differ across regions (*p*-value > 0.05) (Table 2). A nonlinear relationship was observed between plasma retinol concentration and M/P ratio (the power exponent was −1, and *p* for the test of nonlinearity was 0.002), with a breakpoint at 1.00 μmol/L plasma retinol (Figure 2). Below the breakpoint, the M/P ratio, as plasma retinol increased by 1.00 μmol/L, was decreased by 1.69 (95% CI: 0.62, 2.75), while above the breakpoint it was decreased by 0.29 (95% CI: 0.16, 0.42) (Table 3). According to the subgroup analyses, the relationships between plasma retinol concentration and the M/P ratio varied by breastfeeding practices and delivery modes (Figure 3) (Supplementary Table S1).

## 4. Discussion

In this multicenter cross-sectional study involving a relatively large sample of Chinese breastfeeding women, we found that plasma retinol concentration was linearly related with the breast milk, but nonlinearly with the M/P retinol ratio.

Our study found a linear relationship between plasma and breast milk retinol concentrations in women at 42 days postpartum, with an adjusted regression coefficient of 0.34, similar to those from women in other countries [9,10,11]. Two cross-sectional studies conducted among breastfeeding women in Brazil (*n* = 136) and Thailand (*n* = 166) consistently showed a significant regression coefficient of 0.30 [9,11]. The third cross-sectional study conducted among breastfeeding women in Kenya (*n* = 62) showed a significant regression coefficient of 0.26 [10]. Besides the regression analysis, our correlation analysis showed a mildly significant correlation coefficient of 0.17, which was similar to the result of a Kenyan study (r = 0.16) [12], but lower than that of Indonesian studies (r = 0.37 and 0.32) [7,8]. Three Brazillian studies showed a null correlation [13,14,15], likely due to their limited samples size (42 to 72 subjects).

The relationship between maternal and breast milk retinol in breastfeeding women is likely affected by maternal VA intake [26,27]. A study conducted in Indonesian VA-deficient women (*n* = 170) reported a significant relationship (r = 0.30) in women who did not take VA supplements but no relationship in those taking VA supplements [28]. Another study conducted in Cameroonian women (*n* = 162) found that plasma retinol-binding protein, a proxy for plasma retinol, was positively correlated with breast milk retinol only among women whose VA intake were in the bottom or middle tertiles, but not among women in the top tertile [29]. Duration of breastfeeding might also affect the relationship between maternal and breast milk retinol concentrations. A Thai study found a positive relationship among women who breastfed for more than 6 months but not among those who breastfed for less than 6 months [9]. In addition, previous studies that found positive relationships all used mature milk [7,8,9,10,11,12], whereas none of those using colostrum found any significant relationships [16,17,18,19].

The linear relationship between plasma retinol concentration and milk retinol concentration suggests that there might be a diffusion gradient transfer process of retinol from plasma to breast milk. The nonlinear relationship between plasma retinol and the M/P ratio might reflect another transfer process from plasma to breast milk. The M/P ratios were the highest at the lowest level of plasma retinol, then steeply dropped and then tapered off. The breakpoint value of plasma retinol was approximately 1.00 μmol/L. Underneath and above this value, the M/P ratio, as plasma retinol increased by 0.1 μmol/L, was decreased by 0.17 and 0.03, respectively, indicating that the capacity of transferring retinol from plasma to breast milk is stronger in women with a low level of plasma retinol than those with a high level. When the plasma retinol concentration of breastfeeding women is low, maternal liver reserves are drawn upon to compensate for dietary intake inadequacy, and the capacity of transferring retinol from plasma to breast milk are enhanced to allocate retinol preferentially to the breast milk [30]. A study in Kenya showed no significant difference between breast milk retinol concentration in VA deficient and non-VA deficient breastfeeding women [12]. Another study in Zambia [31] showed that some breastfeeding women had adequate breast milk retinol concentration even with low total liver VA reserves. These findings indicated that in the maternal body’s mechanism, it is a priority to supply breast milk retinol to satisfy the demands of the infant as far as possible, even in the case of VA deficiency. This biomedical phenomenon might be crucial to the build liver reserves needed after weaning for infants.

Our study has strengths. Firstly, this is the first study to investigate the relationship between plasma retinol with breast milk retinol and the M/P ratio based on a multicenter cross-sectional design with a relatively large sample of breastfeeding women enrolled from central, southern and northern regions of China. Secondly, the plasma retinol and breast milk retinol were analyzed using standardized HPLC methods, leading to internationally comparable results. Our study has limitations. Firstly, the study participants were all 42 ± 7 days postpartum and from relatively well-nourished urban populations, which limits the generalization of the findings to populations in other lactation periods or from rural areas. Secondly, the detection of plasma retinol and breast milk retinol through retrospective analysis of these biological samples may have limitations compared with real-time detection of biological samples at the time of collection.

## 5. Conclusions

In conclusion, we found a positive and linear relationship between plasma and breast milk retinol that persisted in different regions among Chinese breastfeeding women. We further found a nonlinear relationship between plasma retinol and the M/P retinol ratio, as the capacity of transferring retinol from plasma to breast milk was stronger in women with a low level of plasma retinol than those with a high level. The potential modifiers of the relationship between maternal plasma retinol and breast milk retinol are warranted to be investigated in future studies.

## Figures and Tables

**Table 1:** Maternal and infantile characteristics by regions.

Characteristics	Overall (*n* = 403)		Regions						
			Central (*n* = 135)		Southern (*n* = 134)		Northern (*n* = 134)		*p*-Value *
	*n*	%	*n*	%	*n*	%	*n*	%	
Mothers:									
Age (years)									0.034
<25	60	14.9	13	9.6	29	21.6	18	13.4	
≥25 to 30	251	62.3	91	67.4	81	60.5	79	59.0	
≥30	92	22.8	31	23.0	24	17.9	37	27.6	
Ethnicity									0.036
Han	385	95.5	131	97.0	131	97.8	123	91.8	
Others	18	4.5	4	3.0	3	2.2	11	8.2	
Education level									0.264
College or above	270	67.0	88	65.2	84	62.7	98	73.1	
High school	91	22.6	34	25.2	31	23.1	26	19.4	
Middle school or less	42	10.4	13	9.6	19	14.2	10	7.5	
BMI (kg/m^2^)									<0.001
<18.5	9	2.2	1	0.7	7	5.2	1	0.7	
18.5 to <25	273	67.7	83	61.5	104	77.6	86	67.7	
≥25	121	30.0	51	37.8	23	17.2	47	35.1	
Parity									0.281
Primiparous	343	85.1	119	88.1	109	81.3	115	85.8	
Multiparous	60	14.9	16	11.9	25	18.7	19	14.2	
Gestational age (week)									0.488
<37	16	4.0	6	4.4	5	3.7	5	3.7	
37 to <42	379	94.0	124	91.9	127	94.8	128	95.5	
≥42	8	2.0	5	3.7	2	1.5	1	0.7	
Delivery modes									<0.001
vaginal delivery	242	60.0	112	83.0	79	59.0	51	38.1	
cesarean section	161	40.0	23	17.0	55	41.0	83	61.9	
Breastfeeding Practice									0.193
Exclusively breastfeeding	240	59.6	73	54.1	87	64.9	80	59.7	
Partially breastfeeding	163	40.4	62	45.9	47	35.1	54	40.3	
Infants:									
Sex									0.049
Male	212	52.6	70	51.9	61	45.5	81	60.4	
Female	191	47.4	65	48.1	73	54.5	53	39.6	
Birth weight (g)									0.456
<2500	5	1.2	1	0.7	2	1.5	2	1.5	
2500 to <4000	358	88.8	116	85.9	123	91.8	119	88.8	
≥4000	40	9.9	18	13.3	9	6.7	13	9.7	

* Chi-square test was used to compare percentages by regions.

**Table 2:** Retinol concentrations and M/P ratio and correlation coefficients between plasma retinol, breast milk retinol, and M/P ratio.

	Overall	Regions		
		Central	Southern	Northern
Retinol concentration (μmol/L) in plasma				
Median (IQR) ***	1.39 (1.21, 1.63)	1.50 (1.30, 1.71) ^a^	1.31 (1.10, 1.50) ^b^	1.42 (1.25, 1.67) ^a^
Mean ± SDs	1.44 ± 0.37	1.53 ± 0.35	1.32 ± 0.36	1.48 ± 0.37
Retinol concentration (μmol/L) in breast milk				
Median (IQR) **	1.15 (0.83, 1.49)	1.20 (0.91, 1.57) ^a^	1.20 (0.87, 1.55) ^ab^	1.03 (0.72, 1.40) ^b^
Mean ± SDs	1.22 ± 0.56	1.28 ± 0.54	1.26 ± 0.60	1.10 ± 0.52
M/P ratio				
Median (IQR) ***	0.80 (0.57, 1.12)	0.79 (0.59, 1.05) ^ab^	0.97 (0.68, 1.29) ^a^	0.68 (0.49, 1.00) ^b^
Mean ± SDs	0.88 ± 0.44	0.87 ± 0.38	1.01 ± 0.51	0.77 ± 0.38
Correlation between plasma and breast milk retinol ^†^				
Crude r_s_	0.17 **	0.15	0.18 *	0.24 **
Partial r_s_ ^¶^	0.17 **	0.23 *	0.21 *	0.12
Correlation between plasma retinol and M/P ratio ^§^				
Crude r_s_	−0.34 ***	−0.33 ***	−0.38 ***	−0.25 **
Partial r_s_ ^¶^	−0.33 ***	−0.25 **	−0.33 ***	−0.37 ***

^†^ In the overall analyses and subgroup analyses stratified by regions, crude and partial Spearman correlation coefficients were used to correlate plasma and breast milk retinol. ^§^ In the overall analyses and subgroup analyses stratified by regions, crude and partial Spearman correlation coefficients were used to correlate retinol concentrations in the plasma and milk-to-plasma (M/P) ratio. ^¶^ In the overall analysis, we adjusted for regions, maternal age, ethnicity, education level, BMI, parity, gestational age, delivery modes, breastfeeding practice, sex and birth weight of infants, in subgroup analyses stratified by regions, we adjusted the same confounders except regions. ***, *p* < 0.001; **, *p* < 0.01; *, *p* < 0.05. ^a^ and ^b^ indicate significant difference in concentration (a > b), and ^ab^ indicates non-significant difference.

**Table 3:** Multivariate linear regressions between retinol concentrations in breast milk and plasma, and between M/P ratio and plasma retinol concentration.

Outcome	Exposure	β (95% CI) ^†^	SE	R^2^
Breast milk ^§^	Plasma	0.34 (0.19, 0.49)	0.08	0.16 ***
M/P ratio ^¶^	Plasma	−0.40 (−0.51, −0.28)	0.06	0.22 ***
M/P ratio ^‡^	Plasma ≤ 1.00 μmol/L	−1.69 (−2.75, −0.62)	0.54	0.25 ***
	Plasma > 1.00 μmol/L	−0.29 (−0.42, −0.16)	0.07	

^†^ In the multivariable regression models, we adjusted for regions, maternal age, ethnicity, education level, BMI, parity, gestational age, delivery modes, breastfeeding practice, sex and birth weight of infants. ^§^ Linear regression between the retinol concentration in breast milk and plasma (μmol/L). ^¶^ Linear regression between the M/P ratio and plasma retinol (μmol/L). ^‡^ Linear regression between the M/P ratio and plasma retinol, and plasma retinol as a segmented linear variable with a knot value (1.00 μmol/L). ***, *p* < 0.001. SE, standard error.

## Data Availability

Data described in the manuscript, code book, and analytic code will be made available upon request pending.

## Supplementary Materials

The following supporting information can be downloaded at: https://www.mdpi.com/article/10.3390/nu15245085/s1, Figure S1: Flow Chart of this study. Table S1: Multivariate linear regressions between the M/P ratio and plasma retinol concentration, stratified by breastfeeding practice and delivery modes.
