# Supplementary material for: A Correlation Study of Plasma and Breast Milk Retinol Concentrations in Breastfeeding Women in China
Source: Nutrients. 2023 Dec 12;15(24):5085. doi: 10.3390/nu15245085 (PMC10745653; doi:10.3390/nu15245085)
Supplement: Supplementary file 1 [file nutrients-15-05085-s001.zip › Supplementary Figure S1.pptx]

## Slide 1
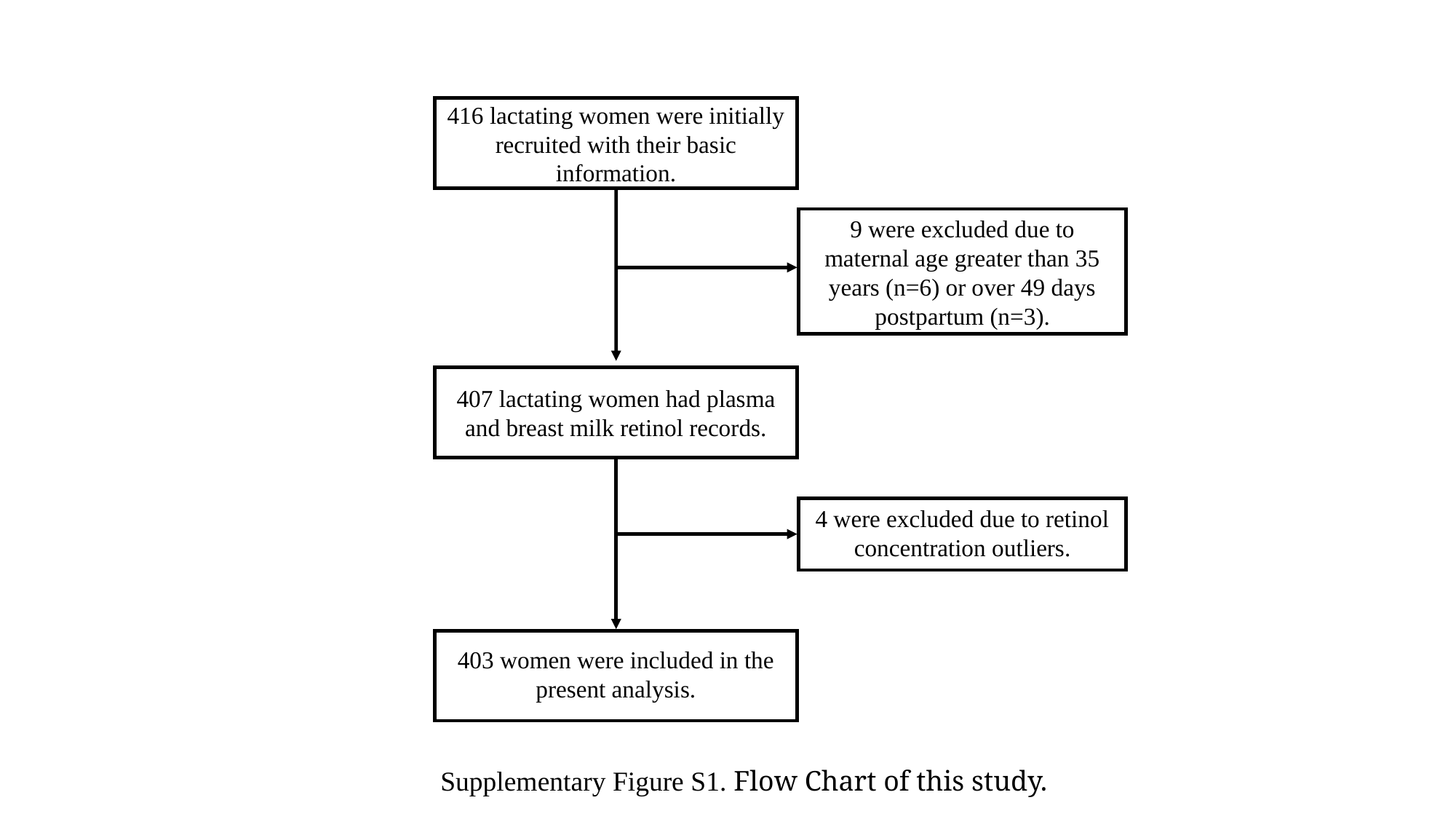

416 lactating women were initially recruited with their basic information.
9 were excluded due to maternal age greater than 35 years (n=6) or over 49 days postpartum (n=3).
407 lactating women had plasma and breast milk retinol records.
4 were excluded due to retinol concentration outliers.
403 women were included in the present analysis.
Supplementary Figure S1. Flow Chart of this study.
