# Supplementary material for: A Correlation Study of Plasma and Breast Milk Retinol Concentrations in Breastfeeding Women in China
Source: Nutrients. 2023 Dec 12;15(24):5085. doi: 10.3390/nu15245085 (PMC10745653; doi:10.3390/nu15245085)
Supplement: Supplementary file 1 [file nutrients-15-05085-s001.zip › Supplementary Table S1.docx]

**Supplementary Table S1.** Multivariate linear regressions between milk-to-plasma (M/P) ratio and plasma retinol concentration, stratified by breastfeeding practice and delivery modes.

| **Outcome** | **Exposure** | **β (95%CI) ^†^** | **SE** | **R^2^** |
| --- | --- | --- | --- | --- |
| Partially breastfeeding | | | | |
| M/P ratio ^¶^ | Plasma | −0.38 (−0.56, −0.19) | 0.09 | 0.23 ** |
| Exclusively breastfeeding | | | | |
| M/P ratio ^‡^ | Plasma ≤ 1.25 μmol/L | −1.20 (−1.69, −0.71) | 0.25 | 0.32 *** |
|  | Plasma > 1.25 μmol/L | −0.15 (−0.36, 0.06) | 0.11 |  |
| Cesarean section | | | | |
| M/P ratio ^¶^ | Plasma | −0.32 (−0.51, −0.14) | 0.09 | 0.22 ** |
| Vaginal delivery | | | | |
| M/P ratio ^‡^ | Plasma ≤ 0.96 μmol/L | −5.00 (−7.61, −2.38) | 1.33 | 0.36 *** |
|  | Plasma > 0.96 μmol/L | −0.30 (−0.46, −0.14) | 0.08 |  |

**^†^** In the multivariable regression models, we adjusted for regions, maternal age, ethnicity, education level, BMI, parity, gestational age, delivery modes, breastfeeding practice, sex and birth weight of infants.

^¶^ Linear regression between M/P ratio and plasma retinol (μmol/L).

^‡^ Linear regression between M/P ratio and plasma retinol, and plasma retinol as a segmented linear variable with a knot value.

***, *p* < 0.001; **, *p* < 0.01. SE, standard error.
